# Optical Fiber, Nanomaterial, and THz-Metasurface-Mediated Nano-Biosensors: A Review

**DOI:** 10.3390/bios12010042

**Published:** 2022-01-14

**Authors:** B. M. Azizur Rahman, Charusluk Viphavakit, Ratchapak Chitaree, Souvik Ghosh, Akhilesh Kumar Pathak, Sneha Verma, Natsima Sakda

**Affiliations:** 1 School of Mathematics, Computer Science and Engineering, University of London, London EC1V 0HB, UK; sneha.verma@city.ac.uk (S.V.); natsima.sakda@city.ac.uk (N.S.); 2 International School of Engineering and Intelligent Control Automation of Process Systems Research Unit, Faculty of Engineering, Chulalongkorn University, Bangkok 10330, Thailand; charusluk.v@chula.ac.th (C.V.); akhilesh.k@chula.ac.th (A.K.P.); 3 Department of Physics, Faculty of Science, Mahidol University, Bangkok 10400, Thailand; rachapak.chi@mahidol.ac.th; 4 Department of Electronic and Electrical Engineering, University College London, Gower St., London WC1E 6AE, UK; souvik.ghosh@ucl.ac.uk

**Keywords:** optical fiber, nanomaterial, metasurface, biosensor

## Abstract

The increasing use of nanomaterials and scalable, high-yield nanofabrication process are revolutionizing the development of novel biosensors. Over the past decades, researches on nanotechnology-mediated biosensing have been on the forefront due to their potential application in healthcare, pharmaceutical, cell diagnosis, drug delivery, and water and air quality monitoring. The advancement of nanoscale science relies on a better understanding of theory, manufacturing and fabrication practices, and the application specific methods. The topology and tunable properties of nanoparticles, a part of nanoscale science, can be changed by different manufacturing processes, which separate them from their bulk counterparts. In the recent past, different nanostructures, such as nanosphere, nanorods, nanofiber, core–shell nanoparticles, nanotubes, and thin films, have been exploited to enhance the detectability of labelled or label-free biological molecules with a high accuracy. Furthermore, these engineered-materials-associated transducing devices, e.g., optical waveguides and metasurface-based scattering media, widened the horizon of biosensors over a broad wavelength range from deep-ultraviolet to far-infrared. This review provides a comprehensive overview of the major scientific achievements in nano-biosensors based on optical fiber, nanomaterials and terahertz-domain metasurface-based refractometric, labelled and label-free nano-biosensors.

## 1. Introduction

Since the 1970s, when the revolutionary advancement in fiber optics technology took place, extensive research has been dedicated towards this area. As a result, the optical fibers broadened their use from optical transmission waveguides in telecommunications to sensing devices for different applications, including monitoring temperature, mechanical strain, refractive index (RI), pressure and concentration of analytes. Recently, optical fibers have become an important part of sensing technology. Their demand as a sensing probe is increasing more specifically in pharmaceutical, clinical, military and industrial applications. The advance features of optical fiber include long interaction length, excellent light delivery, and cost effectiveness. It also has ability not only to excite the target molecules, but also capture the emitted light from the targets, which make its use favorable in biosensing.

Nanotechnology address substances with sizes ranging from 0.1 to 100 nm; nevertheless, due to their small size, these materials differ from their bulk counterparts in terms of electrical conductivity, high reactivity, magnetization, and spectral characteristics. In other words, it is a multidisciplinary field that encompasses physics, electronics, material science, chemistry, and allied engineering fields. It is a recent field of science with numerous applications ranging from energy generation to industrial manufacturing processes to pharmaceutical properties. However, when it comes to nanomaterials, there is no uniform globally accepted definition as different organizations have differing viewpoints [1]. According to the Environmental Protection Agency (EPA) [2], “nanomaterials can demonstrate distinct characteristics different to the identical chemical component in a broader dimension”. The US Food and Drug Administration (USFDA) also defines that “nanomaterials with at nanometer scale of roughly 1 to 100 nm can demonstrate dimension-dependent phenomena” [3]. Nanostructures have also gradually acquired popularity due to their numerous applications, and this has opened new areas for developing optical biosensors. Due to the effectiveness of localized and unlocalized surface plasmon resonance (LSPR), nano-shaped structures and thin films might give a good possibility for molecular-level biosensing applications, as described by Farooq et al. [4]. The real-time monitoring of proteins, toxins, bacteria, glucose, viruses, etc., is highly desirable and significant in cryobiology, food hygiene, microbiological detection, and pharmaceutical research and development. Several optical detection techniques have been explored and reported to date as discussed in the following sections. However, the low-loss silica optical fiber, developed by the Corning Corporation in 1970, has proved its application as a medium of light transmission that was later utilized as a biosensing probe and since then it has gained extensive attention [5]. Metasurfaces are fascinating designs to operate in the THz radiation range according to the corresponding wavelength of THz that is related to large bonding molecules, which are mostly biomolecules such as proteins, RNA, DNA, or antibody–antigen. In this review paper, we discuss varieties of biosensors based on optical fibers, nanoparticles, and terahertz metasurfaces.

## 2. Fiber Optic Biosensors

An optical fiber is a cylindrical waveguide that guides the light within the core of the fiber [6] based on the total internal reflection (TIR) phenomenon. With the advancement of deposition of nanomaterials, the area of optical fiber sensors has expanded in terms of research directions and possibilities. For the development of new sensors, nanostructured thin films and nano-coatings have been applied to a variety of optical fiber configurations. With these combinations, several devices have been fabricated and commercialized to detect multiple physical and chemical parameters, such as temperature, mechanical strain, refractive index, chemical and biological fluids, and concentration of analytes. Recently, optical fibers have become an important part of sensing technology. In the case of sensing, one should exploit the evanescent field of the guiding light. Several methods have been explored to strengthen and utilize the evanescent field to enhance the sensitivity of biosensors, such as tapered fiber, D-shaped fiber, tapered fiber on chip, hetero core fiber, etc. Considering the physical structures and unique light guiding phenomenon, optical fiber-based biosensors can be divided into three categories: (1) conventional optical fiber-based biosensors; (2) grating-based biosensors; and (3) microstructured fiber biosensors.

### 2.1. Conventional Fiber Optic Biosensors

**Figure 1:**
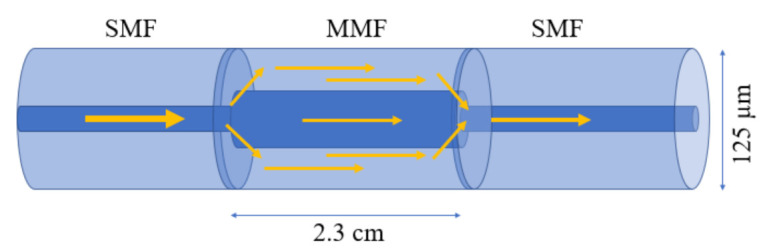
Schematic diagram of a fiber hetro-core structure.

In general, optical fiber is categorized into two types, based on the number of modes propagating through it: (1) single-mode fiber (SMF) and (2) multi-mode fiber (MMF). A SMF allows only one mode to propagate along with the fiber core, because of its small core diameter (8–10 µm). On the other hand, MMF allows multiple modes to propagate along the fiber due to its large core diameter (50–100 µm) [7]. In general, the fiber-based biosensors comprise a thin metallic film or nanostructure along the length of the sensing area to excite the surface plasmon resonance (SPR) or localized surface plasmon resonance (LSPR), which can be later immobilized with various antibodies or sensing materials for target-specific detection [8,9]. The sensing configuration based on the SPR or LSPR effect can be excited from the evanescent field extended into the cladding region and interacted with deposited metals [10,11,12]. After decades of investigations, several sensing configurations based on optical fiber have been reported for their real-time application in biosensing. For biosensing purposes, the hetero-core sensing configuration has been exploited with several fruitful results for G proteins, refractive index monitoring, etc. [13,14,15]. The hetero-core sensing configuration favors the excitation of cladding modes by stubbing an SMF between two MMF or vice versa [16]. Recently, Lokendra et al. utilized a similar sensing configuration coated with gold nanoparticles (AuNPs), polyvinyl alcohol (PVA) stabilized silver nanoparticles (PVA-AgNPs), and graphene oxide (GO) to diagnosis the L-cysteine content in human urine in 2020 [13]. In the reported sensor, AuNPs and PVA-AgNPs are utilized to excite the LSPR phenomenon and the additional layer of GO is used to provide the larger binding sites and surface area to detect L-cysteine molecules in urine samples. The sensing configuration is illustrated in Figure 1. The work compared two configurations: the optical fibers coated with AuNPs/GO, and PVA/AgNPs/GO. The sensor achieved a good limit of detection (LOD) of 152.5 µM and 126.6 µM, with a sensitivity of 0.0012 nm/µM and 0.0009 nm/µM, respectively. Although the sensor performs well, poor sensitivity may limit its application because of low-light–matter interaction in this configuration. Hence, the hetero-core sensors have not been much utilized in biosensing applications. The light–matter interaction was later improved by several other configurations, such as uncladded/etched fiber and U-shaped fiber sensors.

**Figure 2:**
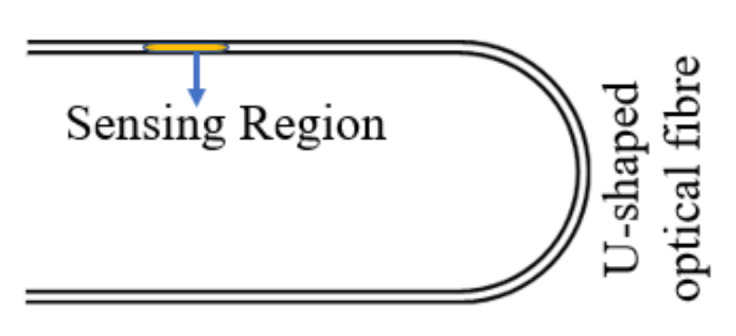
Schematic of a U-shaped fiber bend.

Uncladded/etched fiber has been considered as another popular configuration in biosensing applications. The structure can be fabricated by removing a small section of fiber cladding and then the sensing material is deposited to facilitate as a sensing head [17,18,19]. When the light propagates along the core, some part of the field may extend outside of the core into the cladding region, which is known as evanescent field. It can interact with the sensing material to detect the surrounding variations. In 2013, Jie et al. reported gold nanosphere (GNSs) and gold nanorod (GNRs)-coated optical fiber biosensor based on the LSPR phenomenon, which was later immobilized with human IgG in order to detect various concentrations of anti-human IgG [20]. The GNS- and GNR-deposited fiber sensors demonstrate an average LOD of 1.6 nM for anti-human IgG. Recently, in 2018, Qian et al. reported an SPR-based uncladded optical fiber biosensor [21]. The reported work utilized plastic fiber in which the cladding was removed from a 5 mm long section. A thin bimetallic layer of Cr (2 nm) and Au (50 nm) were deposited on an uncladded portion, which works as sensing region. Later on, the Au surface was functionalized using phenylboronic acids (PBA) by self-assembly method to detect glycoprotein. The LOD achieved for concanavalin A was as low as 0.29 nM by L-PBA self-assembled monolayer modified fiber sensors. Compared to the uncladded/etched fiber structure, the evanescent field of the sensing structure was enhanced by bending the fiber into a U-shape structure near the sensing region. It is considered as potentially popular in SPR-based fiber biosensors with increased sensitivity and LOD [22,23,24]. In a recent article, Jinchuan et al. reported a fluorescent immunosensor for microcystin-LR based on U-shaped optical fiber [25]. The fluorescence sensitivity of this U-shaped probe with light-sheet skew ray excitation is 16 times more than that of collimated skew ray excitations. Integrating into this feature, authors have developed a highly sensitive and real-time sensor for microcystin-LR detection based on the indirect competitive immunoassay principle. The real environmental water samples spiked with microcystin-LR were observed by the immunosensor with good recovery rates between 85% and 112%. The schematic diagram of the developed sensor is shown in Figure 2.

In addition to the discussed experimental realization, various researchers have performed detailed numerical investigations over various sensing configurations in order to provide highly sensitive and portable devices. Some researchers have utilized D-shaped SMF-based optical fiber sensors along with metallic nanowires, thin films, graphene layers, nano-columns, etc. [10,11,26,27,28].

### 2.2. Grating-Based Biosensors

**Figure 3:**
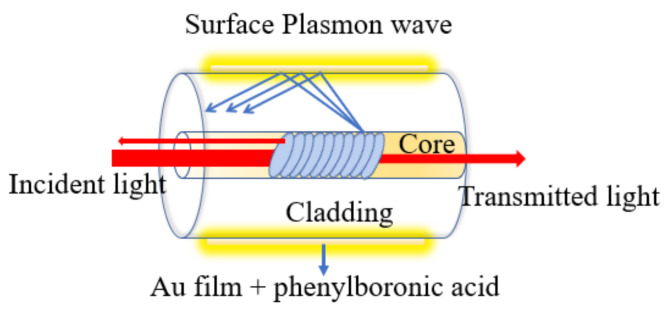
Schematic diagram of the working principle of a titled fiber grating sensor.

The first grating in germanium-doped core was fabricated and reported by Hill et al. in 1978 [29]. The grating structure was made by laser lithography technology to facilitate the permanent period change of the refractive index in the fiber core. After several decades of investigations, the fabrication of grating-based fiber and its commercialization has been achieved. The sensing principle of grating-based fibers can be defined as the grating period, grating length, and the effective refractive index of such fibers that are affected by the variation in the surrounding media. The change in the outer environment leads to the change in its resonance condition; consequently, the variation in resonance wavelength takes place [30]. Based on the property of these gratings, it can be classified into three categories: (1) fiber Bragg grating (FBG), (2) long-period fiber grating (LPFG), and (3) tilted FBG (TFBG) [31]. The grating-based fibers have become increasingly attractive to biomedical applications in the last 20 years because of their unique features of compact size, immunity to electromagnetic interferences, biocompatibility, highly sensitive, in situ monitoring, and multiplexing capability [32,33,34,35,36]. Owing to their design of label-free monitoring of refractive index (RI), grating-based sensing configurations, such as LPG, etched FBG, and TFBG, have attracted extensive attention in order to develop chemical and biosensors [37]. Among several configurations, some of the FBG-based sensors have been tested in order to improve thrombin biosensing [38]. Thrombin is involved in both normal and abnormal coagulations. The normal concentration of thrombin in blood ranges from nanomolar to low micromolar during the coagulation process [39,40]. Thrombin leads to several disorders, including thromboembolic disease, atherosclerosis, inflammatory disease, and cancer, hence its monitoring in the blood is highly required for both research and clinical use [41]. In addition to the FBG thrombin sensor, other grating-based biosensors have been reported utilizing LPFG and TFBG. In 2012, Saurabh et al. exploited the role of LPFG to develop the first bacteriophage-based detection of *Escherichia coli* (*E. coli*) [42]. The reported device was based on the spectral interrogation technique for the real-time monitoring of the binding of *E. coli* bacteria on the immobilized fiber surface by measuring the shift in the resonance wavelength of output spectra form LPFG. The LPFG surface was immobilized with T4 bacteriophages to bind *E. coli* and observed a good shift in resonance wavelength varying from 2 to 4 nm for *E. coli* concentrations range from 10^3^ cfu/mL to 10^9^ cfu/mL, with an excellent experimental accuracy greater than 99%. Later in 2016, L. Marques et al. reported a streptavidin protein sensor using LPFG functionalized with a PAH/SiO_2_: AuNPs thin film [43]. The adopted LPFG was fabricated by considering a length and grating period of 40 mm and 110.7 μm, respectively. The reported configuration achieved a sensitivity of 6.88 nm/(ng/mm)^2^ with theoretical LOD of 19 pg/mm^2^ at 23 °C and 50% relative humidity. In 2017, Zhang et al. proposed a label-free TFBG biosensor utilizing the SPR phenomenon to monitor glycoprotein [44]. The sensing configuration utilizes a thin gold film (50 nm) immobilized with phenylboronic acid as a specific recognition of glycoprotein, as shown in Figure 3. The sensor exhibits a linear response between amplitude and the concentration. It shows that the amplitude decreases with an increase in the concentrations of glycoprotein. The maximum sensitivity of 2.857 dB/(mg/mL) was achieved along with LOD of protein of 15.56 nm.

The TFBG shows excellent sensing response when integrated with SPR or LSPR phenomenon. Although, in 2018, Mederic et al. developed a highly sensitive TFBG-based biosensor against cytokeratin without any additional metallic coating [45], the reported device was focused on the internal property of the sensor at near-infrared wavelength by immobilizing the receptors directly on the fiber surface. The functionalization method plays a major role in the sensitivity enhancement of the sensor. Several authors consider the electrostatic adsorption of anti-CK17 to be the fastest and the most effective grafting strategy. Covalent bonding illustrates a larger shift between 10^−10^ and 10^−9^ g/mL of CK17 in comparison to the progressive response for adsorbed antibodies. The etching of TFBG was introduced later in the same year in order to enhance the sensitivity of the sensor. Marzhan et al. reported the increase in sensitivity from 1.25 nm/RIU to 23.38 nm/RIU for a broad range of refractive indices varying from 1.3418 to 1.4419 [46]. Their proposed sensor was examined for the monitoring of thrombin–aptamer interactions based on silane-coupling surface chemistry, with thrombin concentrations varying from 2.5 to 40 nM. The functionalization of the etched TFBG provided a competitive sensing platform for biochemical monitoring and exhibits a high sensitivity varying from 2.3 to 3.3 pm/nM specifically for thrombin detection. Furthermore, the reported sensor shows excellent sensitivity without any additional metallic depositions on the fiber surface, which makes the sensor cost effective compared to other available devices. In 2020, a similar etching technique was utilized on FBG by Kavitha et al. to improve the LOD by depositing the reduced graphene oxide (rGO) on the etched portion for the detection and quantification of dsDNA [47]. In that work, authors reduced the diameter up to 10.1 µm, and immobilized rGO on top of it to reach an overall diameter of 13.92 µm. The developed sensor achieved a good LOD of 261.87 pg/µL with a linear response for dsDNA varying between 1 ng/µL and 50 ng/µL; the etched surface of the FBG illustrates a good sensing response with excellent stability and repeatability. Additionally, the authors used UV absorbance spectroscopy to determine the actual quantity of attached DNA on the surface of the sensor.

Recently, in 2021, Maxime et al. drew a TFBG in multi-mode fiber and demonstrated their relative potential for refractometry and biosensing purposes [48]. In the reported work, the authors compared the sensing response of the sensor by introducing TFBG in both SMF and MMF with a gold coating on the surface. Gold-coated MMF-TFBGs exhibit a refractometric sensitivity of 124.89 nm/RIU, which is ∼22% enhanced in comparison to SMF-TFBGs. Additionally, both configurations were functionalized with anti-human epidermal growth factor receptor-2 (HER2) aptamers to evaluate their sensing performances for the real-time detection of breast cancer biomarker. Both TFBG configurations illustrate an equivalent response to the increasing concentrations of HER2 proteins, ranging from 10^−12^ to 10^−6^ g/mL. TFBG-based biosensors are in situ, cost-effective, and simple to realize in comparison to conventional biosensing platforms without compromising the sensitivity of biological sample measurements.

### 2.3. Microstructured Fiber-Based Biosensors

**Figure 4:**
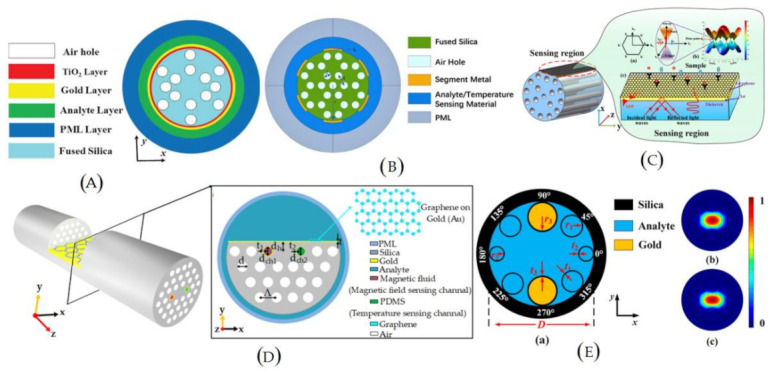
Schematic of MOF-based biosensors: (**A**) bimetallic-coated SPR refractive index sensor; (**B**) outer metal coated PCF sensor; (**C**) graphene–Au-coated SPR sensor; (**D**) multi-parameter D-shape PCF sensor; and (**E**) negative curvature PCF sensor for low refractive index sensing.

Microstructured optical fiber (MOF) is a new kind of fiber that is different in design compared to traditional fibers. It contains a periodic structure in the cladding (air holes) instead of core and cladding or a similar defect in the core to obtain a performance different than that of traditional fibers. Several MOF-based biosensors have been reported in the past decades due to the availability of longitudinal pores that perform as tiny sample chambers and provide the effective RI for light confinement simultaneously [49,50,51]. The schematics of novel MOF-based biosensors can be seen Figure 4. Similar to traditional fibers, the MOF is made of a single material and has an appropriate cross-sectional design. As an experimental demonstration, in 2015, Nguyen et al. developed and reported a biosensor using the multi-mode interference effect in exposed core fibers [52]. In the reported sensor, biotin molecules were functionalized to the exposed surface of fiber to capture streptavidin. At the highest intensity frequency of the spectrum, 0.625 nm^−1^, the sensitivity was estimated to be 667 nm/RIU. The platform proposed in that experiment is feasible and can provide a potential label-free biosensing detection method for clinical research. Later in 2016, Gao et al. developed an air-hole-collapsed sensing configuration to detect DNA hybridization and methylation [53]. The sensor was based on the Michelson interferometry technique formed in the photonic crystal fiber (PCF). This sensor comprised a micro-hole fabricated in the collapsed region using femtosecond laser micromachining, which combined the tunable mode coupler and optofluidic channel. In the reported sensor, the probe ssDNA is bound to the surface of the optical fiber through the poly-L-lysine layer. It is detected by hybridization of the short probe ssDNA with the complementary target ssDNA. Experimental results exhibited a good LOD of 5 nm.

In 2017, Wu et al. reported a numerical and experimental investigation over a side polished PCF sensor [54]. To realize the sensor, a thin gold film (45 nm) was deposited on the polished facet hexagonal structured PCF. The sensing response of the sensor was performed for a short range of analytes varying from 1.33 to 1.34 and achieved a high sensitivity of 21,700 nm/RIU. Simulation results of the reported work show that it was difficult to improve the sensitivity of the sensor by varying the duty ratio, the lattice pitch of PCF, and the polishing depth. In 2019, Hu et al. reported a new kind of liquid crystal biosensor based on the Mach–Zehnder phenomenon to monitor the enzymatic reactions of penicillinase [55]. The sensing head was fabricated by stubbing a tapered MOF between two SMFs, which was later coated with 4′-pentyl-biphenyl-4-carboxylic acid (PBA)-doped 4-cyano-4′-pentylbiphenyl (5CB). At the same time, the experiment obtained a local linear fit with a tapered MOF diameter of 50 μm and a pH range of 7.8 to 6.6. The pH measurement sensitivity was 1.21459 with good linearity of 0.97761. In addition to the above experimental demonstrations, several groups performed detailed numerical investigations for different novel sensing configurations, such as D-shaped photonic crystal fiber (PCF), mono-rectangular core PCF, twin-core PCF, etc., to enhance the sensitivity, improve the figure of merit, and provide the user-friendly interface [50,56,57,58,59,60].

## 3. Nanomaterials for Biosensing

Nanomaterials are described as substances with at least one exterior dimension of between 1 and 100 nanometers. According to the European Commission’s definition, at least half of the particles in a size distribution must have a particle size of 100 nm or less. Nanomaterials are also being explored for defense use, with one example being the employment of movable pigment nanoparticles to provide a better kind of camouflage by injecting nanoparticles into the material of soldiers’ uniforms. Nanomaterials’ features, notably their size, provide a variety of benefits over composite counterparts, and their adaptability in terms of the ability to customize them for specific applications further enhances their applicability. Their high porosity is another benefit, which raises the demand for their application in a variety of sectors. Nanomaterials can improve the efficiency and cost effectiveness of conventional energy generation systems, such as solar panels, while also opening new ways to collect and store energy in the energy industry. In the electronics and computer industries, nanomaterials are expected to bring a variety of benefits, such as their usage allowing for greater atomic-level precision in the fabrication of electronic circuits, aiding in the creation of a wide range of electronic devices. Nanomaterials’ enormous surface-to-volume ratio is particularly important in the medical industry, since it allows cells and active substances to connect. Consequently, the chances of successfully combating numerous diseases are increased. Additionally, researchers have invented different sensors that can identify deadly pathogens utilizing a variety of nanomaterials. A detailed description about the nanomaterials is given below.

### 3.1. Classification of Nanomaterials

**Figure 5:**
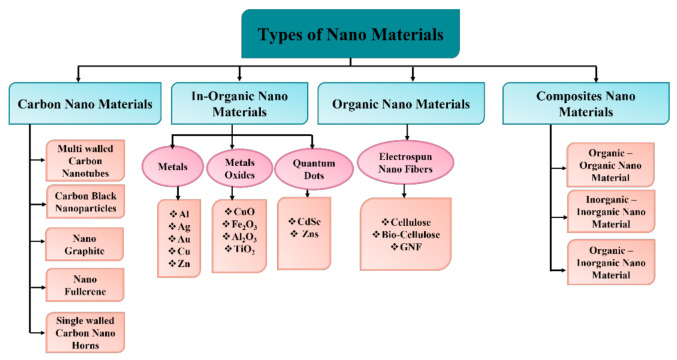
Classification of nanomaterials [68].

Nanomaterials are classified mainly in four groups as (1) carbon nanomaterials, in which the carbon compound is present and appears in geometries, such as hollow wires, ellipse, spheres, and holes. The group of these nanomaterials includes diamonds, fullerenes, carbon nanotubes (CNTs), nanofibers (C60, C80, and C240) and onions [61,62]. Since the 1990s, they have spurred advancements in physics, electronics, optics, mechanics, biology, and medicine due to their remarkable characteristics and distinct carbon hybridization status (e.g., sp2 and sp3 hybridization) [63]. However, the group of (2) inorganic nanomaterials consists of a variety of nanometals and its oxides/alloys. Different kind of metals can be used in this category, such as gold (Au), silver (Ag), copper (Cu), zinc (Zn), and aluminum (Al). TiO_2_, CuO, Fe_2_O_3_, SiO_2_ and Al_2_O_3_ can be used as oxide nanoparticles. The group of (3) organic nanomaterials are mostly made from organic materials except carbon and inorganic nanomaterials (discussed above). The utilization of noncovalent (weak) interactions for the self-assembly and design of molecules helps to transform the organic NMs into desired structures, such as dendrimers, micelles, liposomes, and polymer nanomaterials [64]. Finally, the group of (4) composite nanomaterials are mainly a multiphase type of nanomaterials. It is also known as hybrid materials, where one phase of nanoparticles can either combine with another phase of the material or with a larger bulk material. These are considered as the most complicated structures since they consist of organic metal frameworks. These nanomaterials are basically a combination based on carbons, metals, ceramics, and different kinds of polymers. There are many, with numerous morphologies and uses, such as gold–zinc oxide (Au/ZnO) floral rods used for fetoprotein detection, titanium dioxide–cerium oxide (TiO_2_/CeO_2_) nanowires used as low-cost high-performance catalyst systems, and molybdenum trioxide-reduced graphene oxide (MoO_3_-rGO) hybrid nanobelt utilized as cathode material for lithium batteries [65,66,67,68,69]. A detailed description of the nanomaterials is shown in Figure 5.

### 3.2. Nanomaterial Classification Based on Dimensionality

**Figure 6:**
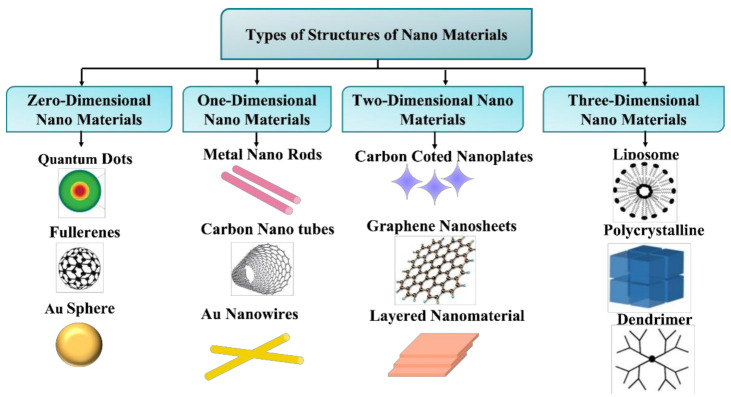
Classification of nanomaterials based on their dimensions, based on [72].

The fabrication of traditional nanostructures is now accelerating and will continue to assist various nations’ economic growth. Many kinds of nanoparticles and nanostructures have been discussed in recent works, and many more are expected to arise in the future. As a result, the necessity for their classification has intensified. In 2000, Gleiter et al. proposed the first idea for nanostructure categorization [69]. In that study, nanomaterials were categorized based on their crystalline morphologies and chemical nature. However, the Gleiter method was incomplete since the dimensions of the nanoparticles and nanostructured materials were not considered [70]. Furthermore, zero- and one-dimensional (0D, 1D) structures, such as fullerenes and nanotubes, were not considered in Gleiter’s approach. As a result, instead of 12 classes, there were 3 classes and 4 types in each of them under this system. Nanostructures should be distinguished from nanostructured materials because the former (nanostructures, NSs) are defined by their shape and geometry, whilst the latter (nanostructured materials, NSMs) are defined by their composition. As a result, nanostructures should be categorized correctly based on one of these characteristics, specifically, dimensionality, which is a broad natural property that includes size, shape, and appearance. There are an unlimited number of shapes that can be chosen for bulk 3D materials. Because of their low dimension, the atomic difference between some nano shapes of the same dimensions can be ignored throughout the transition into the nano world. As a result, the number of nano structured classes becomes limited. This raises the issue of current nanostructure categorization. In the year of 2007, Pokropivny and Skorokhod developed an innovative scheme of classification for nanomaterials that includes recently developed composites, such as 0D, 1D, 2D and 3D nanomaterials [32]. The 3D units are excluded because, except for the 3D matrix, they cannot be utilized to construct low-dimensional NS 3D structures; on the other hand, they only can be termed NSMs if they include 0D, 1D, and 2D NSs. This is precisely the scenario examined by Gleiter in his categorization of NSMs [71]. The brief classification of nanostructures based on dimensions is presented in Figure 6.

According to “surface engineering,” size and shape have now become key components in determining the characteristics of nanoscale materials. Pokropivny et al. showed the 36 categories of nanostructured material in Figure 5, whereas Gleiter et al. discusses only 4 classes [69], albeit the missing 32 classes are merely new exact categories that pertain to the fascinating realm of nanotechnology [71]. From the above discussion it can be concluded that geometry always plays a vital role in the physics of the nanostructure. If we focus on Einstein’s concept of general relativity, it is easy to understand that “physics is the combination of geometrical and physical rules”. This idea may be restated in the context of nanotechnologies as follows: “nanophysics is the field that is made of the surface and size of nanostructures along with the key features of physical laws present in materials”. To understand clearly the most common dimensionality of nanostructures, we characterized these in four branches: zero-dimensional, one-dimensional, two-dimensional, and three-dimensional nanostructures [64].

### 3.3. Nanomaterial Classification Based on Physical and Chemical Properties

Nanostructures have some unique physical and chemical characteristics, such as high-surface-to-volume ratio, and good mechanical and optical conductivity, and high chemical reactivity makes them suitable candidates for a wide range of applications. Some of their most important properties are discussed in the following sections.

#### 3.3.1. Thermal Properties

The thermal characteristics of nanoparticles are influenced by a variety of parameters that are barely detectable in bulk materials. Surface characteristics, topological nanostructures, and quantum or conventional size effects heavily influence heat conduction in nanomaterials, resulting in carrier dispersion and localization that are otherwise absent or undetectable in bulk materials [73]. In much simpler language, thermal properties are those of a substance that are correlated towards its thermal conductivities. In other words, these are just the characteristics that a material exhibits whenever its temperature is increased. Thermal characteristics are part of the broader subject of the physical properties [74,75,76,77]. In metallic nanomaterials, electrons and phonons seem to be good energy carriers, but phonons are the dominant energy carriers in non-metal crystalline materials [78]. The wave vectors and mean free path of phonons transport a substantial amount of energy at the nanoscale level in nanostructured materials. As a result, determining the mean free path distribution of phonons is critical to comprehend the heat conduction properties of phonons as transporters.

#### 3.3.2. Mechanical Properties

Nanomaterials have unique mechanical characteristics to find new applications in a variety of areas, including thermodynamics, surface research and engineering, and nanostructures. To determine the actual mechanical nature of nanoparticles, several mechanical characteristics, such as Young’s modulus, toughness, stress–strain, adherence, and resistance, can be investigated. Surface coating, clotting, and greasing, in addition to these factors, are size dependent, which helps to enhance the mechanical properties of nanoparticles [79]. Controlling the mechanical performance of nanoparticles, as well as their reactions with the coated surface and other factors, is important for improving the surface quality and optimizing material removal [80,81]. There are many fascinating challenges and mechanical forces that occur when we shrink in size as observed by Feynman [82]. These interactions between the surface of nanoparticles generate several forces, such as Van der Waals (vdW) forces, electrostatic force, and electrical double layer (EDL) force, capillary force and solvation, structural and hydration forces. Van der Waals (vdW) forces are feeble interactions between nanoparticles that play a vital influence in the mechanical behavior of the nanoparticles. This kind of mechanical forces are further distributed into three categories. The first one is defined as the orientation force (the Keesom force) [83], which is the consequence of the interactions between the persistent dipolar movement of polar molecules. The second one is the induction force (the Debye force) [84], which is caused by the interaction of the polar molecule’s persistent dipole moment with the produced dipole moment. Finally, the last is the dispersion force (the London force) [85,86], which arises through induced instantaneous dipole polarization and is found in a wide range of polar and non-polar molecules. Electrostatic and electrical double layer (EDL) forces occur when any nanoparticle comes into contact with water or any liquid that has a different refractive index and is generally charged, and the electrostatic repulsive force prevents them from aggregating. The charging or discharging of the nanoparticles’ surface in the liquid has three main factors [87], such as (1) the ionization or dissociation of functional groups on the surface; (2) the deposition or bonding of ions from the liquid onto a formerly uncharged surface; and (3) whenever two different surfaces are very close together, electrons can bounce over from one to the other. The electrical double layer is formed when the surface electrons are fairly distributed by an oppositely charged ion layer in the liquid at a distance from the surface. Helmholtz was the first to explicitly propose the EDL, who used the basic molecular capacitor model to calculate the charge distribution in the solution [88]. The production of liquid menisci (also known as the meniscus force) is the primary cause of capillary force, which was initially recognized by Fisher [89] and Haines [90]. Standard capillary force and lateral capillary force are the two forms of capillarity forces [91]. Many researchers have also provided detailed studies of the typical capillary force on the soil’s granular substances [92,93,94,95,96]. Besides vdW and EDL forces, additional forces, such as solvation, structural, or hydrating forces, come into consideration whenever two materials or nanoparticles comes into contact with any liquid at an extremely near distance (the distance being smaller than a few nanometers). At tiny separations, electromagnetic forces can be monotonically repulsive, over time attracting or oscillating, and they can be considerably larger than vdW or EDL forces. If indeed the solvent or water molecules are organized by the surfaces, solvation, structural, or hydrating forces (in any liquid) occur between two nanoparticles or nano surfaces [97].

#### 3.3.3. Magnetic Properties

Magnetic nanoparticles have attracted the attentions of researchers in a diverse variety of fields, including heterogeneous and homogeneous catalysis, biomedicine, magnetic fluids, storage systems, magnetic resonance, and environment monitoring, such as water treatment. According to the research, nanoparticles perform best when their size is less than 10–20 nm [98]. The magnetic characteristics of nanoparticles is efficiently regulated at a small scale, making these particles valuable and useful in a variety of applications [98,99,100,101]. The magnetic property of nanoparticles is caused by their irregular electrical dispersion, and these characteristics are also reliant on the synthetic technique, and they may be prepared using a variety of methods, including solvothermal [102], co-precipitation, micro-emulsion, thermal breakdown, and flame spray formulation [103].

#### 3.3.4. Optical and Electrical Properties

**Figure 7:**
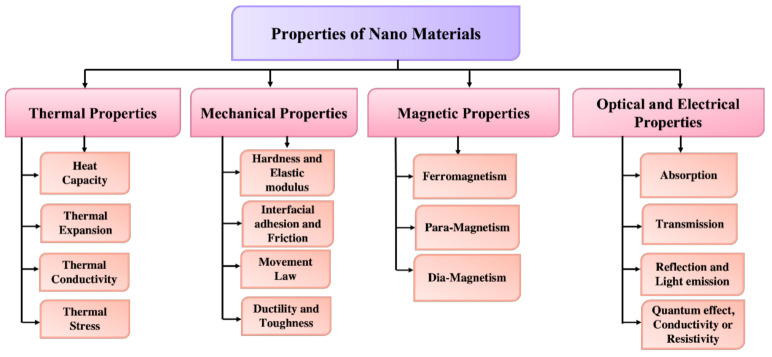
Classifications of nanomaterials based on their physical and chemical properties.

The optical and electrical characteristics of nanoparticles are more intertwined. Noble metal nanoparticles, for example, have size-dependent optical characteristics and have a significant ultraviolet absorption band that is absent from the bulk material’s spectra. The localized surface plasma resonance (LSPR) occurs whenever the incident photon frequencies are constant with the collective excitation of the electron density. The wavelength selection absorption has exceptionally large molar vibrational coefficient resonant frequency, i.e., ray light scattering with efficiency comparable to that of ten fluorophores and enhanced local electromagnetic fields closer to the surface of NPs that enhances spectrometry, which results in LSPR excitation. The dimensions, geometry, and interparticle spacing of the NPs, as well as its own dielectric characteristics and those of its surrounding environment, such as the substrate, solvents, and adsorbates, are all known to affect the peak wavelength of the LSPR spectrum [104]. The rusty shades found in blemished glass doors/windows are caused by gold colloidal NPs, whereas Ag nanoparticles are usually yellow. The number of electrons on the surface of these nanoparticles can easily move through the nanomaterial. Silver (Ag) and gold (Au) have a mean free path of 50 nm, which is larger than these materials’ NPs. As a result, following light interaction, there is no anticipated dispersion from the bulk; rather, they enter a standing resonance state, which is fundamental for LSPR in these nanoparticles [105]. Figure 7 shows the schematic of the properties along with its branches.

## 4. Application of Nanomaterials in Biosensing

Nanotechnology has gained its popularity in recent years due to its extremely efficient performances in absorption, scattering, extinction, and transmission/reflection at nanoscales; nevertheless, there remain numerous unexplored potentials. Although Synge introduced the idea of near field microscopy in 1928, it was not explored due to the manufacturing limitations of the time. Bailey and Fletcher secured a patent on Electromagnetic Wave Converters in 1973, which was the first discovery of tiny antennas that is comparable to modern-day nano-antennas [106]. Later, in 1985, Wessel published an idea for high electric field confinement caused by tiny nanometallic particles, which can be seen using scanning microscopy [107] and explored the significance of these nanosized particles’ surface plasmon resonance. Alvin M. Marks described a super sub-micron electron beam writer for direct light-to-electric-current conversion in 1989 [108]. In recent years, research on nanoparticles has remained popular, and a range of articles have been published that demonstrate the remarkable efficiency in terms of implementations. This systematic review discusses the influence of several nano-structural dimensions and their characteristics for an array of applications. In 2004, Atay and Song [109] created circular periodic arrays of gold structures with significant resonance and far-field patterns. Likewise, Lahiri et al. created asymmetrically split ring resonators for biological material detection [110]. Nanostructures are also employed in surface enhanced Raman spectroscopy (SERS), which may respond for a broad region of the electromagnetic spectrum [111]. Additionally, paired structures containing different shapes, such as the bowtie [112,113,114,115], nano-disks [116,117,118], nanorods [114,119,120,121], and nano elliptical-shaped antennas, have also been reported [122,123] in visible regions in recent years, which can be studied for many applications, such as bio-medicine, communications, and solar cell and water quality control by varying the geometries and surrounding medium; however, there are several areas still left to be considered.

In 2003, Michael R. Beversluis designed surface plasmon enhanced transitions of gold nanospheres for the visible and infra-red photoluminescence continuum [124] and, parallelly Alexandre Bouhelier simulated elliptical cluster of gold particles for optical devices [125]. Nanospheres have also been used for secure and controllable drug delivery systems, hyperthermia, and static/dynamic thrombolysis assessment [126], and used for the drug delivery and therapeutic approaches that are particularly effective as targeting agents in tumor-bearing subjects [127]. It also showed promising results for psoriasis treatment, methotrexate drug delivery, and topical therapy in psoriasis patients [128]. In 2017, these nanospheres have also treated cervical cancer, which can help to decrease the death rate among women [129]. In the following years, Sarah Elisabeth Ochmann reported single-molecule-based point-of-care diagnosis for Zika virus detection [130]. Gold nanorods were designed in 2016 by Priscila Falagan-Lotscha for biological applications, such as cytotoxicity detection [131], colorimetric determination of hypochlorite from water [132], fluorescence enhancement [133], the killing of tumor cells via photothermal ablation [134], strain sensing applications [135] and solar cell applications [136]. Gold nano-disk has been used for hydrogen sensing [137], PSA cancer marker detection [138], energy harvesting and spintronics/magnetics, biosensors [139], optical switching [140], medical diagnostics drug delivery or chemical sensing [141], and other sensing applications [142]. Maura Cesaria designed nano holes for nano-optical transducers sensing and integrated/multiple detection of lab-on-a-chip devices using unconventional lithography [143]. Bowtie antenna has shown highly encouraging results in terms of biological sensing and nano-optic applications [144], bioinspired surfaces and dielectric metamaterials [145], and polarimetric optical biosensing [11]. Elliptical nano shaped antennas showed promising results for near-field scanning optical microscopy [22,146]. Researchers have also designed a gold nano star for a number of applications, such as HeLa cells transfection with PGFP under optimized conditions [147], singlet oxygen production [148], early cancer detection [149], tumor detection and killing [150], and photothermal therapy/targeted drug delivery and anti-tumor/anti-bacterial devices [151]. There is another nano cubic structure that shows its significant role in some applications, such as phenolic biosensors [152], autoantibody detection from body fluids samples [153], cell imaging of human liver cancer cells (QGY) and human embryonic kidney cells [154], biology and medicine [155], nanoscale galvanic replacement reactions [156], anticancer natural product [157], plasmonic refractive index sensing [158], photoacoustic imaging-guided radio/photodynamic/photothermal synergistic therapy [159] and photoacoustic imaging of tumor protease capturing the vibrational fingerprints of lipid molecules [160,161]. Some antenna geometries have been also studied in the form of gold nanoplates for monitoring pH in saliva [162]. Yamin Yang et al. has designed gold nano rings for photodynamic cancer therapy [163]. An asymmetric-split ring resonator has been used for AFM imaging and plasmonic detection by increments in sensitivity with an order of magnitude over the non-resonant structures and water treatments [164,165]. Butterfly nano antennas have also been designed for orbital angular momentum (OAM) applications [166]. Diamond-shaped antennas have been fabricated for biotechnology [167] and mushroom-shaped antenna showed good opportunities for refractive index sensing [168]. Dumbbell- and parabolic-shaped structures showed impassive results for photovoltaics, electroluminescence, non-linear optics, and plasmon excitations [169,170]. The above literature review and Table 1 shows the promising application, advantages, methods and limitations of strong resonance and field confinement through numerical modeling and experimental investigations during the last few years.

## 5. Application of Metasurfaces for Biosensing

The whole electromagnetic spectrum, covering from very low radio frequency to very high cosmic ray, has a wide range of valuable applications, particularly in the microwave, infrared, and visible bands. On the other hand, despite the extensive attention paid to some of these spectra, there is a small gap between the infra-red and microwave, known as the “terahertz (THz) gap” [175], which has often been ignored. Not only the popularity of microwave and infra-red bands has caught the attention of researchers, but also the present difficulties in THz generation and detection are another factor that has, to date, made THz less attractive. However, recent developments of the technology to have better sources and detections have created a renewed interest in this frequency band.

Terahertz radiation is the spectrum that has the frequency, as the name suggests, in the order of 10^12^ Hertz, but the whole band is not formally defined and is mostly considered from 0.3–30 THz, but it has also been considered from 0.1–10 THz [176]. In the THz region, some unique properties make the THz spectrum suitable for sensing applications. THz is non-ionizing radiation [177,178,179,180], as it does not have enough power to ionize or damage the biological tissue or living cells, which can reduce the possibility of developing cancer. Therefore, THz radiation is safer compared to that of X-rays. Secondly, with the frequency of THz being around 10^12^ Hz, its wavelength is around hundreds of microns, which is a good match to the bonding length of many biomolecules and is sensitive to the weak resonance, such as hydrogen bond and van der Waals force [181,182,183,184,185]. Therefore, THz can be useful in offering new ways to identify a large molecule, such as proteins, RNA, DNA, or antibody–antigen. Finally, THz is strongly sensitive to the water molecule and the hydrogen bond [186,187,188]. As a cancer cell is an abnormal cell with different water content levels compared to a normal cell, the THz band can play an important role in differentiating and detecting the cancer cell without ionizing the molecules or causing cancer [189,190,191].

One of the challenges for biosensing applications is to establishment of measurements from a small amount of the sample. To work with such a small amount of sample, a thin film-based device can be more suitable. The thin film of metallic structures possesses unique light-guiding or scattering properties compared to their bulky counterparts. In addition, due to a thin film in a sensing region, a strong evanescent light tail can easily reach the sample. Thus, a small amount of sample volume is sufficient for the devices with a thin film [192]. However, as mentioned before, the THz regime has difficulty in having suitable sources and detectors, where the metasurface can provide a solution. The metasurface is an artificial and engineered material that can tailor its electromagnetic properties by incorporating a periodic pattern [193,194,195,196]. The required transmittance, reflectance or absorptance properties needed in the THz regime can possibly be designed as a periodical pattern with a specific shape and size. The thin-film technology is applied in its manufacturing as it needs the periodic patterned structure to create such metasurfaces in which the top layer is often considered as the thin film [197,198,199,200]. In this section, some of the metasurfaces that have been designed to operate in the THz regime are discussed with a focus on biosensing applications, including the detection of proteins, cancer, viruses, DNA, and biomaterials.

**Figure 8:**
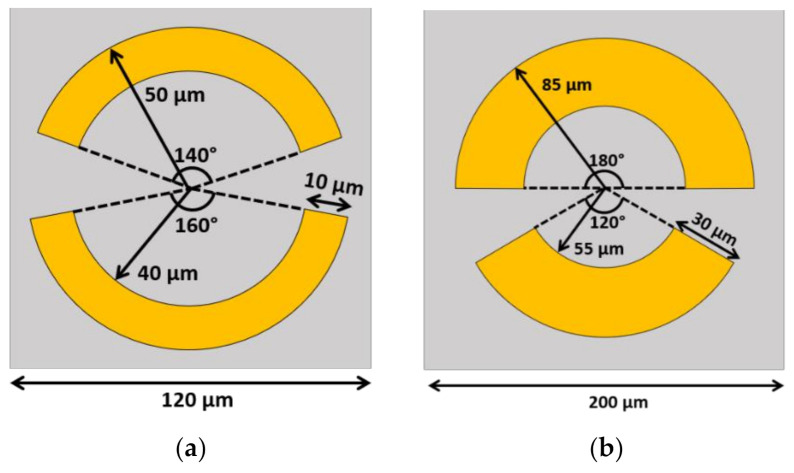
The schematic of the asymmetric split-ring resonators. (**a**) The unit cell that proposed in [197] to detect the protein. The central angles are 140 and 160 degrees with the inner and outer radii as 40 and 50 µm, respectively. (**b**) The unit cell proposed in [198] to detect DNA.

Ruijian et al. [201] proposed a metasurface with an asymmetric split-ring resonator pattern to detect proteins. The unit cell consists of two layers of a periodic pattern of 200 nm thick copper on 25 μm polyimide substrate with 120 μm periodicity. The periodic pattern is a split-ring with an outer radius of 50 µm and 40 µm inner radius, as shown in Figure 8a. Two gaps were introduced to the ring and located with the central angle at 140° and 160°. The numerically simulated and experimentally measured results agree very well on the transmission spectrum in the operating frequency from 0.6–1.8 THz. The transmittance profile provides two resonant dips, the first one located at 0.81 THz with anti-parallel current density distribution. The second dip is located around 1.13 THz with a dipole-like parallel current flow between the top and bottom split arcs. In order to use this metasurface to detect proteins, the refractive index of the material above the unit cell is changed from air (*n* = 1) to a range of *n* = 1.3 to 1.8 to match the refractive index of the protein. The numerical results show both dips are shifted to lower frequency as the refractive index increases. The sensitivity of the first dip is 160 GHz/RIU and the second dip is 140 GHz/RIU. The sample was prepared as the protein A/G (the recombinant fusion of protein A and protein G) and protein A/G+IgG (protein A/G and a goat anti-mouse immunoglobulin G). With 1 mg/mL sample on the fabricated periodic pattern, the transmission shows the shift of both dips. The first case is the difference between the bare metasurface and the protein A/G, when the first dip shifted around 8 GHz to a smaller frequency and the second dip shifted around 11.7 GHz. For the protein A/G+IgG, the sensitivity is higher. The first peak is shifted by 17.6 GHz to the left when it is changed from protein A/G to protein A/G+IgG, while the second peak is shifted around 52.9 GHz. This proposed structure offers a promising opportunity for protein-sensing applications in the future.

Ruiyun Zhou et al. [202] proposed DNA detection by using another complementary asymmetry split-ring (CASR). The unit cell length is 200 µm with a split-ring that has an outer radius of 85 µm and a 55 µm inner radius, as shown in Figure 8b. Two gaps are introduced at the central angle of 120° and 180°. A 100 nm thick gold (Au) pattern was deposited on top of the 1000 µm thick quartz dielectric layer. On the top of the Au periodic pattern, a 1 nm graphene monolayer was added to enhance the sensitivity of the device. Numerical simulations were carried out by using the lumerical FDTD model that shows the transmittance spectrum between 0.28–0.88 THz. The transmittance profile shows two resonance peaks at 0.39 THz and 0.65 THz. The experimental results also show two resonant peaks. To understand more about the effect of the graphene on the metasurface, the Fermi level of the graphene was varied from 0−100 meV according to the Kubo equation. The transmission levels of the two peaks decrease following the reduction in the Fermi level. In the former peak at 0.39 THz, the transmittance decreases from 0.89 to 0.13, and in the latter peak at 0.65 THz, the transmittance decreases from 0.89 to 0.23. In order to evaluate the sensitivity of measuring DNA, CASR was modified to fit with the DNA probe to recognize the specific DNA. The target DNA sequence of *E. coli* O157:H7 is measured by varying its concentration from 0.1–100 µM. The measured transmission increases level when the concentration of the DNA increases. Especially in the area around 0.4–0.6 THz, the sensitivity is higher as demonstrated by the bigger change for each concentration step. In summary, this study proposed a novel way for real-time and label-free biosensing. The sample preparation is also simple, which is more convenient compared to the conventional method.

**Figure 9:**
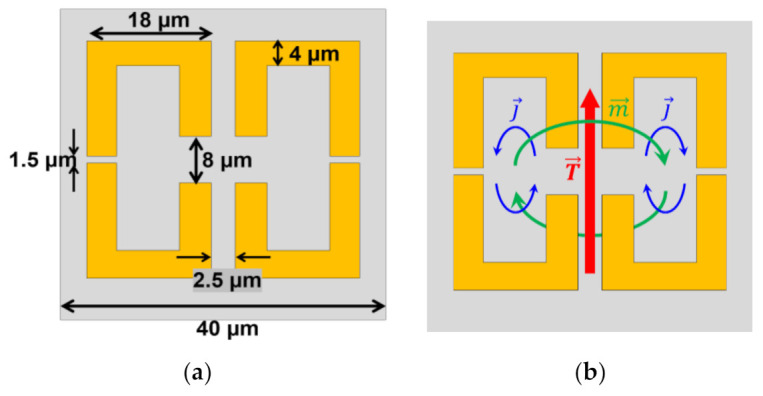
(**a**) The schematic of the metasurface proposed from [199]. The pattern shows two split-ring resonators with big and small gaps and faced against each other. (**b**) The diagram shows how toroidal dipole ($\vec{T}$) can be induced from the magnetic dipole ($\vec{m}$) and the magnetic dipole is generated from current density ($\vec{J}$).

The THz regime is strongly sensitive to the presence of water molecules, which offers the possibility to identify a cancer cell by exploiting the difference in the water content between normal and cancer cells. Chiben Zhang et al. [203] reported the identification of lung cancer cells by using metasurface operating in the THz band. The structure was made from a pair of split-ring resonators (SRRs) placed by facing the gap closed to each other with a 2.5 µm separation, as shown in Figure 9a. Additionally, on the opposite side of the ring, a small gap is also introduced to the structure. The big gaps are of 8 µm and the small gaps are of 1.5 µm, with a 4 μm wide SRRs on the 18 µm long side. The periodicity of the unit cell is 40 µm. The metasurface is made from gold (Au) placed on a 500 µm thick quartz glass substrate. Two SRRs facing each other induce a toroidal dipole, which can enhance the electric fields perpendicular to the SRRs. The magnetic current is excited in each loop of the SRRs, but in the opposite direction. The magnetic dipole loop inducing the magnetic current is placed between the two SRRs. The magnetic dipole loop causes the toroidal dipole along the axis between two SRRs, as shown in Figure 9b. The operating frequency is 1.0 to 3.0 THz. The TE and TM modes of the incident field were investigated by varying the incident angle. The TE mode provides one main resonant dip at 2.39 THz at a zero-incident angle. However, when the incident angle increases to 30°, the resonant dip at 2.39 THz remains similar, but a tiny dip appears at around 2.17 THz. As the incident angle increases further, the transmission amplitude decreases. The first dip at 2.17 THz is a magnetic dipole. The loops of both SRRs are rotated in the same direction, which can be classified as a normal magnetic dipole. By observing the current density of the second dip at 2.39 THz, SRRs show the opposite direction of the surface current, which confirms that it is a toroidal dipole. For the TM mode, the toroidal dipole also occurs at 2.39 THz, but this mode is insensitive to the incident angle as the angle increases up to 30°. To investigate the sensitivity of the metasurface to the biomolecule, the refractive index of the surrounding area was changed from 1.1 to 1.9 to observe the impact. The transmission dip shows a shift of the dip to a lower frequency when the refractive index increases. The sensitivity was reported as 485.3 GHz/RIU. The effect of height and dielectric dissipation are also investigated, and further details can be found in [203]. In the experimental part, to measure lung cancer, three different types of lung cancer were considered, i.e., Calu-1, A427, and 95D. Three different concentrations of the cancer cells were prepared, at 200, 400, and 600 cells/mm^2^ for all three types. For the Calu-1 cells, the different concentrations of the cells show a noticeable shift. The higher concentrate provides more shift to the smaller frequency. The presence of the A427 cancer cells with different concentrations also caused a redshift in the transmission spectrum. There is a difference between 200 and 400 cells/mm^2^, but there is a redshift when the concentration is 600 cells/mm^2^. Finally, for the 95D, there is distinguishable redshift when the concentration of the cells takes place compared to the bare surface. It was suggested that, by considering the shift of frequency and the dip of transmittance, different types of cancer can be distinguished.

**Figure 10:**
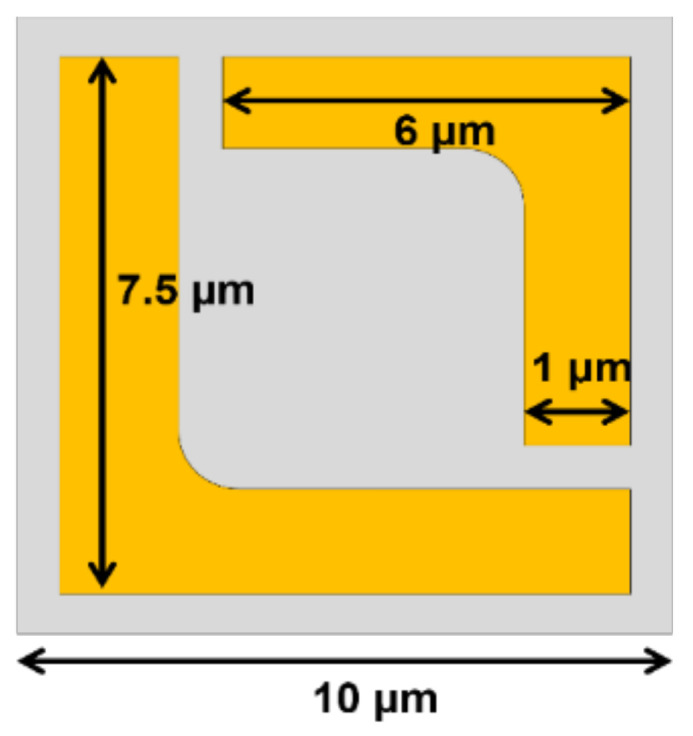
The schematic unit cell proposed from [204] to detect viruses. The periodic pattern is composed of the combination of large L and small L.

Another important application is virus sensing as they can cause so many diseases that can lead to severe health crises and pandemics, such as COVID-19. Unfortunately, to detect viruses by using conventional technique is very time-consuming and also needs a specialist to interpret the results. Masharif Amin et al. [204] suggests a novel way to sense the virus by exploiting sensitive polarization properties to improve the performance. The principle behind the sensitivity of polarization is the localized surface plasmon resonance (LSPR), which normally occurs in the conventional metasurface. However, designing the periodic pattern of metasurface with chiral characteristic can enhance the sensitivity of polarization of such novel metasurfaces. The researchers propose the unit cell as a split-ring with two L-shape graphene sheets. As shown in Figure 10, two L-shapes are slightly different in dimension in order to be combined into a rectangular shape. The former L-shape has a leg length of 7.5 µm and the latter L-shape has a 6 µm length with the width of the split-ring as 1 µm. The graphene metasurface is placed on a 15 µm thick quartz substrate. The periodic pattern repeats every 10 µm in both the x and y directions to create the metasurface. The linearly polarized incident field is numerically investigated for the proposed metasurface from 1.0 to 2.0 THz. The simulation results show two distinct resonant frequencies, which are classified as the left-handed circular (LHC) and right-handed circular (RHC) polarizations. The polarization extinction ratio (PER) was calculated from the reflectance coefficient of the Jones matrix. The first resonant frequency is located at 1.15 THz, which is the dip of the PER and belongs to the RHC. The second resonance peak is at 1.46 THz. The contour plot shows the path of the vector following the LHC. In order to calculate the sensitivity of the virus detection, the numerical investigations not only considered the real part of refractive index, but also its imaginary part (extinction coefficient) to study the effect of the index change to the PER. The results represent both parameters noticeable change the PER. However, to identify the type of the virus, three almost similar influenza viruses were chosen to be investigated, i.e., H1N1, H5N2, and H9N2. The refractive indices (both real and imaginary parts) presented in a reference paper were used as input data to determine the virus variance. By considering the PER spectrum for three viruses, the differences can be observed from the patterns. There are changes in both shifts of the resonance frequency and the magnitude of PER. Moreover, as the designed plasmonic metasurfaces are sensitive to the polarization angle, the orientation angle of the reflectance coefficient can identify the three influenza viruses by using this proposed design.

## 6. Conclusions

In summary, the employability of optical fiber, nanomaterials, and metamaterials in biotechnology is revolutionizing the field of biosensing. This has widened the spectrum of sensing technology due to its smaller footprint, high accuracy, high throughput, and impressive detection limit. However, despite the high sensitivity and low detection limits, selectivity and cross-sensitivity are the underlying challenges of concern. These can be mitigated with the synthesis of new biorecognition, nanocomposite elements, efficient transducing devices, and signal processing algorithms. The benefits of optical transducers include the requirement of a small sample volume, fast detection, small response time, and repeatability. Fiber optic biosensors of different types, such as conventional single and multi-mode, grating-based and specialty microstructured biosensors, attract a lot of interest in the invasive and non-invasive monitoring of different biomedical parameters. Along with the functionalization with nanomaterials, their broadband operation and structural flexibility made the fiber biosensors a suitable candidate for future applications. On the other hand, an on-chip artificially engineered biosensor specially designed for the THz domain is gaining a lot of interest recently. The tunability of the resonance spectrum by changing its physical parameters can mitigate the nonavailability of required sources and detectors in the THz regime. Several recent reports indicate the efficient employment of artificially engineered on-chip metasurfaces in the detection of proteins, immunoglobins, DNA and bulk refractometric variations. Thus, one can foresee the continuous growth of nanotechnology despite all the challenges. Rigorous modeling, optimization, and new fabrication and synthesis processes may lead to improvement in all sensing aspects. This in turn builds up confidence and popularity among researchers to use nanotechnology-mediated biosensors as an efficient tool for biomolecular analysis and healthcare technology.

## Figures and Tables

**Table 1:** A list of nanoparticles and their characteristics in biosensing applications.

Antenna Shape	Material	Method	Limitations	Advantages	Applications	References
Nano Sphere	Gold	Near-Infrared Region, One-Pot Method (Expt.)	It involves a complex experimental system.	Facilitates smart drug delivery system with specific target to thrombus.	Secure and controllable drug delivery system, hyperthermia, static/dynamic thrombolysis assessment.	[126]
Nano Sphere	Gold	UV-vis spectroscopy (Expt.)	It involves a complex experimental system.	Can be used for multiple drug delivery and in vivo evaluation on multiple disease models.	For drug delivery and novel diagnostic and therapeutic approaches, particularly effective as targeting agents in tumor-bearing subjects.	[127]
Nano Sphere	Gold	Spectroscopic techniques (UV-vis and FTIR) and DLS. (Expt.)	It involves a complex experimental system and takes long time.	Methotrexate conjugated with AuNPs shows higher efficiency than methotrexate alone.	psoriasis treatment, methotrexate drug delivery, and topical therapy in psoriasis patients.	[128]
Nano Sphere	Gold	Magnetic Field-Enhanced Radio-Photothermal Therapy (Expt.)	It involves an extraordinary experimental system and takes long time.	It has superb near infrared absorption and excellent superparamagnetic property.	Cervical cancer radio-photothermal treatment, which increases the death rate among women.	[129]
Nano Sphere and Nano Rod	Gold	Chronic Exposure, Acute Exposure, Gene Expression (Expt.)	No significant cytotoxicity was observed.	PEG-coated rods by far induced the largest modifications to gene expression, which has shown that the effect of the NP shape on uptake levels may be highly cell type- and surface moiety-dependent.	Biological applications, cytotoxicity detection.	[162]
Nano Disk and Nano Hole	Gold	Colloidal lithography, FEM (Expt./Sim.)	Requires large areas and low cost is needed for full exploitation.	The realization of large area nanoscale features is important when tunable properties are required.	Biosensors and energy harvesting.	[139]
Disk	Graphene and Silver	Sulfidation (Expt./Sim.)	This work needs advanced experimental facilities.	This work is more affordable and has good optical characteristics.	Sensors.	[142]
Gold Nanoholes	Gold	Unconventional Lithography Techniques (Expt.)	Needs advance experimental facilities.	Compact, low-cost, fully integrated, and multiple-detection lab-on-a-chip devices.	Nano optical transducers in sensing applications, fully integrated and multiple-detection lab-on-a-chip devices.	[143]
Embedded BowtTie Shaped and Hollow Bowtie	Gold	FEM (Sim.)	-	Clearly showed the symmetries of the positive–negative charge distributions.	Imaging, biological sensing, and nano-optics.	[144]
Nano Star	Gold	Plasmonic Optoporation technique, FEM (Expt./Sim.)	It involves high standard laboratories and photopolymerization setup and fluorescence microscopy.	This research improved the capacity of propidium iodide, which was used as a model transfection agent, to enter HeLa cells, as well as the survival of the cells.	HeLa cells transfection with PGFP under optimized optoporation conditions.	[147]
Nanocages and Nanocubes	Gold, Silver	LSPR, Photothermal effect (Expt.)	It involves a complex experimental system.	Even at low Au concentrations, this work showed amazing strong photoacoustic (PA) signals, apparent radiation sensitization, as well as an efficient photothermal effect and ROS generating capabilities.	Photoacoustic imaging-guided radio/photodynamic/photothermal synergistic therapy.	[159]
Gold Nanoplates	Gold	LSPR (Expt.)	It involves a complex experimental system, including scanning electron microscopes (SEM).	This work shows the excellent reversibility for real-time monitoring with short response time.	Monitoring pH in saliva.	[162]
Nano Disk	Gold	LSPR Extinction Spectroscopy Implosion X Nano, FDTD (Expt./Sim.)	Experimental setup is challenging.	This method is label free, less time consuming, simple, highly sensitive and reliable.	PSA cancer marker detection	[138]
Elliptical Shaped Nano Antenna	Gold	FEM (Sim.)	It involves a complex experimental system.	The performance of the sensing device can be improved by altering the geometrical parameters.	Refractive index biosensors.	[171]
Truncated nanocube-shaped	Gold	Simultaneous testing, Morphology control and synthesis (Expt.)	It involves a complex experimental system.	The as-prepared phenolic biosensor can achieve a simultaneous test for trace catechol and hydroquinone at varied working potentials with infrequent interference signal, as well as a high sensitivity, great linear range, and low detection limit.	Phenolic biosensors, optical sensors.	[152]
Gold Nanorods	Gold	Circular Dichroism Spectroscopy (Expt.)	It involves high standard laboratories and experimental system.	It reveals the chiroptical activity of geometrically complex metallic nanostructures, but also establishes valuable design rules for the engineering of next-generation DNA origami-templated nano assemblies with tailorable optical chirality.	Plasmonic chirality of AuNR trimers by resolving them into structurally simpler dimeric components. Dye fluorescence enhancement.	[133]
Gold Nanorods	Gold	LSPR (Expt.)	It involves high standard laboratories and Photopolymerization Setup.	This shows the tumor-specific moieties that have the potential to target tumor tissue to minimize damage to normal tissue.	Conjugation with cytochrome C, kill tumor cells via photothermal ablation.	[134]
Nano Star	Gold	Salt-ageing, Comparative CT method (Expt.)	It involves high standard laboratories and experimental setup and confocal imaging.	This work shows the highly accurate and stable drug delivery.	Anticancer effects.	[149]
Nano Star	Gold	pH-responsive Strategy, Photoacoustic Imaging (Expt.)	It involves a complex experimental system.	The tumor-targeting capacity of pHLIPs and the favorable qualities of GNSs were combined in this study, which might help with tumor imaging and therapeutic research.	Targets the slightly acidic solid tumor microenvironment and tumor accumulation.	[150]
Nano Star	Gold	Seed-Growth Approach, SERS (Expt.)	It needs the high standard procuration of the chemicals.	This research demonstrates the use of a flexible and easy-to-handle starting material to produce sensing, anticancer, and antibacterial devices with good photothermal capabilities.	Photothermal therapy, targeted drug delivery, new sensing, and antitumor and antibacterial devices.	[151]
Nano Cubes	Gold	Michaelis–Menten and Lineweaver–Burk models (Expt.)	It involves a complex experimental system and high standard laboratory facilities.	This study demonstrates how to use both colorimetric and electrochemical readouts to establish a new proof-of-concept platform for autoantibody detection in body fluids samples.	Autoantibody detection body fluids samples using both colorimetric and electrochemical readouts.	[153]
Nano Cubes	Gold	Single-photon excitation, Photoluminescence (Expt.)	It involves a complex experimental system.	This work shows the unique optical properties of the high photoluminescence (PL) of nearly 4 × 10^−2^ quantum yield and a remarkably enhanced extinction band at 544 nm, which are almost 200 times higher than normal gold nano rods.	Cell imaging of human liver cancer cells (QGY) and human embryo kidney cells (293T); photothermal therapy; cell imaging.	[154]
Nano Cubes	Gold	Synthesis of small seeds, short growing process (Expt.)	It shows the complex synthesis of Au nano cubes.	The association was highlighted in this study as a foundation for automated microfluidic synthesis and a variety of applications, such as biosensing.	Sensors in biology, chemistry, and medicine.	[155]
Nano Sphere	Silver	Nucleic Acid Detection Method (Expt.)	It involves a complex experimental system, including confocal measurements and serum preparation equipment.	This work combines the plasmonic signal intensification with signal production by equipping the metal hotspot with a molecular beacon-like structure, resulting in an increased and hence simple to detect signal only in the presence of the specific target nucleic acid.	Single-molecule-based point-of-care diagnosis and Zika virus detection.	[130]
Nano Rod	Silver	SPR, FEM (Sim.)	-	The key benefit of this study is that it demonstrates an adjustable optical spectrum matching to transverse SPR modes while concurrently improving gap enhancement and absorption spectra.	Biosensor and solar cell applications.	[136]
Diamond	Silver	SPR, FEM (Sim.)	-	This study demonstrates the high absorption enhancement factor and efficacy of nanoantenna in the sensing refractive index to identify chemical reagents, solution concentrations, and solution allocation ratios.	Useful for biosensors and biotechnology.	[166]
Coupled Nano Disk	Silver	Raman microscopy, SERS (Expt./Sim.)	This work needs standard experimental setup and laboratories.	This research demonstrates a greater electromagnetic field coupling as well as a very high sensitivity analysis.	Medical diagnostics, catalysis, drug delivery, and chemical sensing.	[141]
Mushroom-Shaped	Gold–Silica	LSPR, FDTD (Sim.)	-	This work demonstrates excellent sensitivity performance.	Refractive index sensing.	[168]
Tubes	Platinum nanoparticles/carbon nanotubes	Electrochemical analysis (Expt.)	This work needs advanced experimental facilities.	Ultrasensitive DNA detection using a sandwich assay with a reduced detection limit, larger linear ranges, and superior stability and repeatability.	Detection of acetylcholine electrochemical DNA biosensors.	[172]
Nano materials	Platinum NP-deposited rGO	Immobilizing glucose oxidase (Expt.)	This work needs excellent experimental facilities.	This work shows high sensitivity with wide linear range, high sensitivity, low detection limit, and fast response time.	Detection of glucose in cherry juice.	[173]
-	Cerium Oxide/Polypyrrole Nanocomposite	Physical adsorption method (Expt.)	It involves a complex experimental system.	This work shows significant selectivity, storage stability, and reproducibility.	Cholesterol sensing application	[174]

Terminologies: Expt.—experiment; Sim.—simulation; SPR—surface plasmon resonance; LIL—Lloyd interferential lithography; ICPE—inductively coupled plasma etching; FEM—finite element method; FDTD—finite difference time domain; LSPR—localized surface plasmon resonance; SERS—surface enhanced Raman spectroscopic; CMOS—complementary metal-oxide semiconductor; DLS—dynamic light scattering technique.

## Data Availability

Not applicable.
